# 脑转移肿瘤细胞及其微环境

**DOI:** 10.3779/j.issn.1009-3419.2016.09.12

**Published:** 2016-09-20

**Authors:** 文 钟, 成平 胡

**Affiliations:** 410008 长沙，中南大学湘雅医院呼吸内科 Department of Respiratory Medicine, Xiangya Hospital, Central South University, Changsha 410008, China

**Keywords:** 肿瘤脑转移, 肿瘤微环境, 星形胶质细胞, 少突胶质细胞, 血脑屏障, 血管形成, Brain metastases, Tumor microenvironment, Astrocyte, Microglia, Blood brain barrier, Angiogenesis

## Abstract

近年来，随着早期诊断的方法的出现及精准治疗的应用，肺癌患者的生存及生活质量都得到很大改善。然而，对于肺癌的脑转移病灶，目前仍缺乏一个理想的治疗方案，严重影响了该部分患者生存状态。了解肿瘤细胞如何在中枢神经系统定植、生长和侵袭等相关生物学行为及其产生机制对预防及治疗肿瘤细胞脑转移病灶具有重大的意义。“种子-土壤”这一假说可以很好的解释这一过程，这一假说的关键即肿瘤细胞可与中枢神经系统微环境各组成之间产生相互适应性变化，正是这种相互作用决定了脑转移病灶的发生发展。本文就脑转移肿瘤细胞、脑转移肿瘤微环境及他们之间的相互作用进行综述，旨在为脑转移病灶的治疗提供新的思路。

## Abstract (en)

Improvements in survival and quality of life of patients with lung cancer had been achieved due to the progression of early diagnosis and precision medicine at recent years, however, until now, treatments targeted at lesions in central nervous system are far from satisfying, thus threatening livelihood of patients involved. After all, in the issue of prophylaxis and therapeutics of brain metastases, it is crucial to learn about the biological behavior of tumor cells in brain metastases and its mechanism underlying, and the hypothesis "seed and soil", that is, tumor cells would generate series of adaptive changes to fit in the new environment, is liable to help explain this process well. In this assay, we reviewed documents concerning tumor cells, brain micro-environments and their interactions in brain metastases, aiming to provide novel insight into the treatments of brain metastases.

在恶性肿瘤的自然病程中，约有20%-40%的患者会出现脑转移病灶^[1]^。而脑转移病灶最常见的来源是肺癌（40%-50%），其次是乳腺癌（15%-20%）、皮肤癌（主要是黑色素瘤）占5%-10%以及消化道恶性肿瘤（4%-6%）^[2, 3]^。近年来由于越来越多的新的治疗方法尤其是精准治疗的兴起，肿瘤患者生存期及生活质量都得到了很大的提高，但由于中枢神经系统（central nervous system, CNS）解剖及生理上的特殊性，各种治疗手段难以达到理想的效果，脑转移病灶因而被称为恶性肿瘤最后的“庇护所”^[2]^。未经治疗的脑转移的患者的中位生存期为1个月-2个月，经过治疗的也仅有6个月左右^[4]^。目前针对脑转移病灶主要治疗手段是放疗、全身性化疗及手术，靶向药物的广泛应用也一定程度改善了脑转移患者的预后^[5]^。然而，恶性肿瘤脑转移患者总体预后仍是难以令人满意的。

肿瘤细胞的转移是恶性肿瘤最为重要的特征之一，转移过程中所涉及细胞内及细胞之间分子机制是十分复杂的，不仅包括肿瘤细胞发生上皮间质转化、循环肿瘤细胞在血管中存活、肿瘤细胞休眠、肿瘤细胞异质性及其干性等变化，还伴随着肿瘤细胞与基质细胞的相互作用、肿瘤相关血管形成等一系列与肿瘤微环境相关事件^[6]^。“种子-土壤”假说即某种特定的肿瘤细胞只有在合适的肿瘤微环境中才能存活，很好地解释了肿瘤特异性转移的发生、发展^[7]^。CNS转移病灶相对于其他部位的转移病灶有其独特性，故明确脑转移肿瘤细胞生物学特性及其与转移灶微环境之间相互作用对于肿瘤脑转移的防治具有重大的意义。

## 1 转移至中枢神经系统的肿瘤细胞生物学特性

在肿瘤转移的过程中，肿瘤细胞为适应远处转移灶的微环境，会发生一系列的生物学特性上的变化^[8]^，Valastyan等^[6]^将此过程归纳为以下五种模式：①肿瘤细胞直接转移形成远处转移灶，不发生改变；②肿瘤细胞转移至远处器官，部分地适应微环境，再产生某些变化以更好适应；③肿瘤原发灶包含多种随机突变（包括具有转移特性的突变）的亚克隆细胞，直接转移至远处器官并形成转移灶；④肿瘤细胞在转移灶已经形成的情况下，原发病灶部分肿瘤细胞可以出现某些改变并进入该转移灶；⑤在癌变过程的早期，部分异型性细胞（quasi-normal cell）可定植在远处器官，并产生突变，再增殖形成转移灶。不论这一过程是发生于肿瘤发生早期、原发灶内、转移灶内或是转移过程中，目的都是为更好适应远处器官的微环境。这种改变可以表现在DNA水平或表观遗传水平上，从而影响肿瘤细胞的表型变化^[9, 10]^。我们将从脑转移肿瘤基因改变、翻译后修饰及代谢特点等方面阐释脑转移肿瘤细胞生物学特性。

### 1.1 脑转移病灶中肿瘤细胞基因改变

Brastianos等^[8]^检测了86例的脑转移病灶及其相匹配的原发灶的病灶基因组点突变及基因拷贝数变异（copy number variations, CNV），通过计算各自的肿瘤-细胞分数（cancer cell fraction, CCF），即通过检测点突变附近的基因拷贝数来估计细胞之间的同源性），描绘出肿瘤细胞的进化树，结果发现，尽管脑转移病灶与原发灶的肿瘤细胞来源于同一祖先，两者却分属于不同的亚克隆。同时，他们还发现，颅内多发病灶的肿瘤细胞亚克隆之间是同源的。其他的一系列相关研究（部分重要基因改变见表 1）也证实脑转移病灶中基因组与原发灶之间存在诸如单核苷酸变异（single nucleotide variation, SNV）、CNV、缺失、扩增等的基因型方面的变化，这些基因组的改变主要涉及多条细胞信号通路的活化、细胞凋亡及细胞的粘附等功能（表 1），并部分解释了肿瘤脑转移形成机制^[11-20]^。

**1:** 脑转移病灶中肿瘤细胞基因改变 Gene profile changes in brain metastases

Authors	Tumor and case number	Matched brain metastasis	Gene profiles	Implication
Ding L, 2010^[11]^	Breast cancer: 1	1	SNV: *NRK*, *PTPRJ*, *WWTR1* CNV: 80.65% overlaps	SNV missense mutation; 19.35% of CNV difference
Brastianos PK, 2015^[8]^	Lung cancer : 38 Breast cancer: 21 Renal carcinoma: 10 Others: 17	86	Mutation: *CDK*, *MCL1* Mutation: *PI3K*/*AKT*/*MTOR*/*PTEN*; *HER2*, *EGFR*, *BRAF*, *MEK*;	Cell cycle proteins; PI3K/AKT/mTOR pathway; HER family; RAF/MEK/ERK pathway;
Bollig-Fischer A, 2015^[12]^	Breast cancer: 10	4	Amplification: *HER2*	HER family
Arai T, 1994^[13]^	Lung cancer: 6 Gastric cancer: 2 Esophageal cancer: 1 Breast cancer: 1	11	Amplification: *HER2*, *EGFR*	HER family
Chen G, 2014^[14]^	Melanoma: 74	30	CNV: generally identical *BRAF*, *NRAS*, *CTNNB1* hot spot mutation: identical	CNV and hot spot mutations: generally identical
Lo Nigro C, 2012^[15]^	Breast cancer: 23	23	Mutation: *TP53*	Anti-oncogene mutation
Wikman H, 2012^[16]^	Breast cancer: 128	15	*PTEN* loss	PI3K/AKT/mTOR pathway
Gaedcke J, 2007^[17]^	Breast cancer: 129	85	CNV: Gain: *EGFR*, *HER2* Loss: *ER*, *PR*	HER pathway; Estrogen and progesterone receptors
Li F, 2015 ^[20]^	Lung cancer: 1	1	CNV: Gain: 1p33-p34, 1q22, 5p13, 14q11 Loss: 3p, 4q31, 5q, 11p15, Xp21-22, Xq21	CNV Gain: leukocyte migration and organ development；hh CNV Loss: proteolysis, negative regulation of cell proliferation and cell adhesion
Preusser M, 2014^[19]^	Lung cancer: 175	175	Amplification: *FGFR1*	FGF/FGFR pathway;
EGFR: epidermal growth factor receptor; HER2: human epidermalgrowth factor receptor-2; ER: estrogen receptor; PR: progesterone receptor; FGFR1: fibroblast growth factor receptor 1; PI3K/AKT/mTOR: phosphatidylinositol-3-kinase/protein kinase B/mammalian target of Rapamycin; CNV: copy number variation.				

研究这一系列变化模式及其产生机制还可为脑转移灶治疗提供有希望的治疗靶点^[8, 14, 19]^。有研究^[8]^发现，在86例肿瘤脑转移的患者中，约有50%的患者脑转移病灶中检测到临床上有治疗意义的突变如人类表皮生长因子受体2（human epidermal growth factor receptor-2, HER2）、表皮生长因子受体（epidermal growth factor receptor, EGFR）、BRAF及AKT等基因的突变，而这些基因在原发灶并未被检出。此外，在乳腺癌脑转移病灶中的HER1及HER2的CNV数较原发病灶有所增加，而雌激素受体（estrogen receptor, ER）、孕激素受体（progesterone receptor, PR）等激素受体的CNV数有所减少^[17]^。同样的，成纤维细胞生长因子受体（fibroblast growth factor receptor, FGFR）扩增、BRAF及NRAS热点突变等基因改变也可以在脑转移病灶中检出^[14, 19]^。

### 1.2 脑转移病灶在表观遗传学上的改变

#### 1.2.1 甲基化

Park等^[9]^对比了黑色素瘤、肺癌、胃癌等多种细胞株在裸鼠脑转移与皮下肿瘤模型中肿瘤细胞全基因组甲基化水平，他们发现脑转移模型中TCF4、PURB、ONECUT2、ESRRG、NFIB及MEF2C等一系列转录因子的甲基化水平有明显的差异，尤其是TCF4这一神经发育相关的转录因子改变最为明显，正是由于这些改变引起脑转移病灶的肿瘤细胞出现独特的基因表达谱。Marzese等^[21]^同样也观察到人黑色素瘤脑转移病灶与颅外病灶有甲基化水平不一致性，如编码多种基因的转录子成分HOX家族成员中HOXD9基因启动子区间甲基化水平明显增高，引起神经发育相关基因的改变。而在乳腺癌脑转移病灶中GALNT9、CCDC8及BNC1等基因的甲基化水平则明显较原发灶增高，而体外沉默上述基因可增强肿瘤细胞的侵袭能力^[18]^。因此，CNS中肿瘤细胞甲基化水平的改变，可能是引起肿瘤表型发生改变原因之一，但究竟是何种因素导致肿瘤细胞的甲基化水平改变的原因尚未完全明确^[9]^。

#### 1.2.2 miRNA

miRNA是长度约19 bp-25 bp的小分子非编码RNA，他们能与mRNA结合后，降解或抑制相应的mRNA，从而影响基因表达，近年来大量的研究已经证实了miRNA是转录后影响蛋白质表达关键因素之一。Zhao等^[22]^通过比较肺癌患者原发病灶与脑转移病灶miRNA水平，筛选出一组下调miRNA如miR-145、miR-214、miR-9及miR-1471等，在其中，他们发现以miR-145下调水平最为明显，而miR-145的下调能促进肺癌A549及SPC-A1细胞株增殖。既往研究^[23, 24]^表明，miR-145可通过参与c-Myc、EGFR、NUDT1表达调节，影响肺癌细胞的增殖与侵袭。miR-145家族中另一成员miR-145-5p也在肺癌脑转移患者中明显下调，因此提高了下游EGFR、OCT-4、MUC-1、c-MYC、TPD52的表达水平，而miR-145-5p下调是由于启动区间甲基化所致^[25]^。miR-200家族的miR-141-3p及miR-200b-3p在脑转移病灶中也较原发灶明显上调，进而下调ZEB2表达，影响肿瘤细胞的增殖、侵袭能力^[26]^。

### 1.3 脑转移病灶中的肿瘤细胞获得神经细胞特性

Park等^[9]^发现肺癌、黑色素瘤、结肠癌的脑转移动物模型中的肿瘤细胞表型上呈现了某些神经元细胞的特性，即谷氨酸信号通路蛋白及神经递质复合物蛋白如SNAP25、SNAP91及BSN等蛋白水平明显增高。同样的，在人乳腺癌脑转移病灶中也发现γ-氨基丁酸（γ-aminobutyric acid, GABA）源的蛋白如GABA受体、GABA转氨酶等表达增高，从而使得肿瘤细胞能在CNS中利用GABA进行各种代谢活动^[27]^。而Nygaard等^[28]^则发现，在黑色素瘤脑转移患者及动物模型中，谷氨酸相关信号通路信号蛋白GRIA2、GRM4及BSN表达增高，促进了肿瘤细胞的生长。此外，还有研究^[29]^表明CNS中的纤溶酶可诱导肿瘤细胞凋亡，而乳腺癌及肺癌细胞株可高表达neuroserpin这一种神经元特征表达的纤溶酶原激活物抑制剂，从而逃避纤溶酶促凋亡的作用。这种转变可能基于高氯的脑组织间液对非神经元细胞具有损伤作用^[30]^，再加上中枢神经充足的神经营养因子、谷氨酸等物质^[31-33]^，肿瘤细胞获得某些神经元特性可能更适合于中枢系统微环境的生存。

### 1.4 脑转移病灶中的肿瘤细胞代谢特点

CNS血供丰富，血流量占全身总量的1/5，因此，其供养、供能也较为充足。由于血脑屏障（Blood Brain Barrier, BBB）的存在，CNS间质葡萄糖较血液浓度低，但亮氨酸、缬氨酸和异亮氨酸等支链氨基酸及谷氨酸含量丰富^[32, 33]^。

相对于肿瘤脑转移侵袭、增殖特性，人们对CNS中肿瘤细胞的代谢特点的所知甚少。Chen等^[34]^对比了脑转移模型与骨转移模型中的肿瘤细胞能量代谢相关蛋白表达的水平，他们发现，不同于常见的肿瘤细胞较多的依赖无氧代谢，CNS中的肿瘤细胞三羧酸循环-氧化磷酸化活跃，并伴有肿瘤细胞磷酸戊糖途径的活化，并可借此机制介导了肿瘤细胞对某些抗代谢化疗药物如D-2-脱氧葡萄糖的耐药。Chen等^[35]^则发现具有CNS转移特性的乳腺癌细胞对低糖耐受能力较其母代细胞更强，并表达了更多的谷氨酸脱氢酶、酮酸脱氢酶以利用环境中的谷氨酸及支链氨基酸。

脂质代谢方面，Chen等^[34]^发现脑转移模型中动物模型中的脂肪酸β氧化相关酶谱较骨转移表达升高，而人的乳腺癌脑转移组织芯片显示，乙酰辅酶A氧化酶-1及脂肪酸合成酶表达水平较其他部位转移灶高，提示脑转移病灶中脂质合成及分解代谢均较活跃^[36]^。

## 2 肿瘤中枢神经系统微环境

脑转移病灶微环境相对于其他组织有着其独特性：①具有BBB、血脑脊液屏障，起到生物屏障作用；②缺乏淋巴细胞、巨噬细胞等免疫细胞，小胶质细胞充当了重要的免疫反应角色^[37]^；③缺乏成纤维细胞等间叶组织，而富含星形胶质细胞及少突胶质细胞；④高表达CXCL-12^[38]^、neuroserpin^[29]^、neutropin^[31]^等CNS特异性的分子。至于颅内微环境对于肿瘤脑转移病灶扮演何种角色目前仍存在争论^[39]^，既往研究^[40-42]^表明，从脑转移模型中分离的肿瘤细胞或与体外CNS微环境共培养获得的肿瘤细胞均较原细胞株有更强增殖、侵袭及转移能力，但另有研究^[29]^则显示星形胶质细胞可分泌纤溶酶原激活物促使肿瘤细胞的凋亡，不利于肿瘤细胞的生长。下面，我们将从构成CNS微环境各成分及其与肿瘤细胞相互作用来探讨颅内微环境对肿瘤细胞的作用。

### 2.1 星形胶质细胞

星形胶质细胞（astrocyte, AST）是CNS中除神经元外最为丰富的细胞。AST在受到刺激情况便可激活，形态上变得肥大，并伴随着AST活化特异性标志胶质纤维酸性蛋白（glial fibrillary acidic protein, GFAP）表达增高。AST具有支持神经细胞、营养神经组织、维持CNS稳态、构成血脑屏障等功能，并能在神经系统受到损伤后起到修复作用。作为CNS微环境最为重要的组分，AST在肿瘤脑转移病灶形成中起到重要的作用^[43]^。

AST活化后可分泌多种细胞因子影响肿瘤细胞的增殖、侵袭、转移能力。有研究发现AST可通过分泌基质金属蛋白酶2（matrix metalloprotein 2, MMP2）及MMP9，清除肿瘤细胞表面及周围基质成分，促进肿瘤细胞的侵袭及转移能力^[44]^，MMP是调节肿瘤微环境最为重要的蛋白酶之一，除了发挥蛋白酶的降解基质、促进侵袭功能之外，MMP2及MMP9可通过激活转化生长因子-β（transforming growth factor-β, TGF-β）调控细胞生长，通过VEGF调节血管生成等功能^[45]^，相关的临床资料^[46]^也表明MMP2表达阳性的脑原位或转移肿瘤的患者的生存期更短。在黑色素瘤脑转移的研究中发现，AST可在黑色素瘤细胞刺激下产生白介素3（interleukin-3, IL-3）、CD40L、CXCL12及IFN-γ等多种细胞因子，其中IL-23则刺激肿瘤细胞产生MMP-2，从而促进肿瘤细胞增殖^[41]^。肿瘤细胞与AST通过细胞因子网络相互作用机制是非常复杂的，有研究表明，CNS中的肿瘤细胞可分泌巨噬细胞迁移抑制因子（macrophage migration inhibitory factor, MIF）、IL-8及纤溶酶原激活物抑制因子1（plasminogen activator inhibitor-1, PAI-1）激活AST，激活的AST又可分泌IL-6、肿瘤坏死因子-α（tumor necrosis factor-α, TNF-α）及IL-1β促进肿瘤细胞的增长，但IL-6R表达却有所下调^[47]^，这与Sierra的结果^[48]^有部分差异。然而，AST除促进肿瘤细胞生长之外，也有研究证实AST对部分的肿瘤细胞的生长可起到抑制作用，主要机制在于CNS中的纤溶酶使得AST细胞膜上的FasL脱落形成分泌型的FasL引发肿瘤细胞凋亡^[29]^。

最新的一项研究^[49]^表明，AST产生外泌体与肿瘤细胞融合后，外泌体内含的的miRNA（主要是miR-19a）可使得肿瘤细胞发生PTEN低表达丢失，进一步活化PI3K/AKT/mTOR通路，促使肿瘤细胞分泌趋化因子2（chemokine ligand 2, CCL2），募集IBA-1表达的髓性细胞，从而促使肿瘤增殖，这一过程特异地发生在CNS中，肿瘤细胞与肿瘤相关成纤维细胞（cancer associated fibroblast, CAF）共培养并未出现此现象。这项发现意义在于可以很好地解释脑转移细胞往往都会出现特异性的PTEN的低表达，从而引起PI3K/AKT/mTOR信号通路过度活化这一现象^[8, 16, 49, 50]^。外泌体是细胞外直径约40 nm-100 nm的小囊泡，可由肿瘤细胞、神经细胞、淋巴细胞及肿瘤细胞等细胞产生，广泛存在于多种组织间液中，其囊泡内含有蛋白质、miRNA、mRNA、脂质等多种细胞成分，作为细胞外的膜性结构可参与细胞之间信号传递，在肿瘤血管形成、转移等过程中起到了重要作用^[51, 52]^。外泌体及其内涵物可以作为肿瘤细胞与其微环境相互作用的途径，进而影响肿瘤细胞的生长，这一发现为脑转移病灶提供一个新颖的治疗靶点，引发了众多关注，外泌体及微囊泡也成为近期肿瘤领域研究热点之一^[53, 54]^。

AST还具有保护肿瘤细胞免受化疗药物细胞毒作用的功能。这种保护机制可能是基于AST与肿瘤细胞的直接接触及间隙连接通信（gap joint communication, GJC）起作用的，但成纤维细胞却不能起到类似的作用^[55, 56]^。AST与黑色素细胞瘤细胞接触后，细胞间的接合素43（connexin 43）的GJC作用可使黑色素瘤细胞免于化疗药物诱导的细胞凋亡^[55]^。此外，AST与肿瘤细胞的直接接触可促使肿瘤细胞分泌IL-6及IL-8，使得AST产生内皮素1（endothelin 1, ET1），ET1与肿瘤细胞的内皮素受体（endothelin receptor, ETR）结合之后可激活AKT及MAPK通路影响下游BCL2L1、TWIST1及GSTA5表达使得细胞免受化疗药物的影响^[56-58]^。类似的，Murphy等^[59]^则在发现connexin 43可通过活化AKT/AMPK/mTOR信号通路诱导恶性胶质瘤对替莫唑胺耐药。但也有研究^[60]^表明，睾丸癌细胞之间却能通过connexin 43之间的信号通路增强化疗药物的细胞毒作用，推测GJC可以传递某些小分子物质诱导肿瘤细胞凋亡。由此可见，肿瘤细胞与微环境、肿瘤细胞与肿瘤细胞之间特定的GJC信号分子可以影响肿瘤细胞在CNS等微环境下对化疗敏感程度，阻断或活化GJC之间的信号传导，对于增强化疗药物效果可能有一定的临床意义。

AST作为微环境的一个关键因素，可与肿瘤细胞相互作用后通过分泌细胞因子网络、直接接触及外泌体等多种途径影响脑转移的肿瘤细胞生物学行为，两者之间作用网络复杂，部分机制尚未完全明确，需得到进一步的阐释^[53, 59]^。

### 2.2 小胶质细胞/巨噬细胞

正常的CNS缺乏淋巴细胞、巨噬细胞等常见的免疫细胞及相应的淋巴管道，小胶质细胞在CNS免疫反应中扮演了重要的角色。小胶质细胞属于单核-巨噬细胞系统，激活后甚至难以从形态学及分子标志上来和循环中的巨噬细胞相区分，而异种共生的动物实验表明，转移瘤模型中的小胶质细胞/巨噬细胞多来源于循环中的单核巨噬细胞，颅内原有的小胶质细胞仅占少数^[61]^。因此，有部分文献将CNS中激活的小胶质细胞直接称之为小胶质细胞/巨噬细胞^[62, 63]^。

免疫系统在肿瘤发生与发展中有着重要的地位。肿瘤中的巨噬细胞主要可分成两型，即M1及M2型，其中M2型单核巨噬细胞表面抗原为CD163及CD204，通过分泌Arginase、IL-10、脂多糖（lipopolysaccharide, LPS）、干扰素γ（interferon-γ, IFN-γ）及转化生长因子-β1（transforming growth factor-β1, TGF-β1）等细胞因子促进肿瘤生长，而M1型则高表达诱导型一氧化氮合酶（inducible nitric oxide synthase, iNOS），并分泌IL-1、IL-12、NO、TNF-α，具有杀伤肿瘤细胞作用^[63, 64]^。Wei等^[63]^回顾了小胶质细胞/巨噬细胞在胶质瘤中的作用，小胶质细胞/巨噬细胞在其中的作用倾向于M2表型，并可与胶质瘤细胞相互作用，促进胶质瘤细胞侵袭及血管生成，但其抑瘤作用仅表现在协同CD8^+^ T细胞、ADCC及少量的M1表型的细胞毒作用这几个方面。小胶质细胞/巨噬细胞作为CNS中免疫功能最为重要的一环，明确脑转移肿瘤中小胶质细胞/巨噬细胞分化类型及其产生机制对明确CNS中免疫系统与肿瘤细胞之间关系有着重要的意义。

体外研究表明，LPS可激活小胶质细胞，促使其向M1型分化，并分泌NO及TNF-α等细胞因子杀伤肿瘤细胞^[61, 65]^，然而，低浓度的小胶质细胞培养上清液却具有促肿瘤生长作用^[65]^，实际上，在肺癌脑转移的病理组织中，尽管肿瘤及周围高表达iNOS，但TNF-α表达量却较低，围绕肿瘤组织的主要是一层厚的IBA-1阳性小胶质细胞/巨噬细胞，这表明在脑转移病灶的不同部位，小胶质细胞有着不同的分化及功能^[65]^，这一功能的差异取决于小胶质细胞/巨噬细胞本身受到微环境的刺激^[63]^。除了典型的M1及M2分化之外，有研究发现乳腺癌细胞刺激下的小胶质细胞可通过非经典的Wnt通路活化通路，促进乳腺癌细胞侵袭及生长^[66]^。证据还来自于Rietkötter等^[67]^的实验，他们发现不同于外周血中的单核/巨噬细胞，小胶质细胞促进肿瘤生长、侵袭作用并不依赖CSF-1的激活作用。

小胶质细胞/巨噬细胞在脑转移病灶中表型变化及相关机制仍有较多空白，以小胶质细胞/巨噬细胞为靶点在治疗肿瘤中远没有当下热门的免疫治疗取得的成果明显。虽有体外实验表明用唑来膦酸可促使CNS中小胶质细胞/巨噬细胞发生表型变化发挥抑制肿瘤侵袭作用^[68]^，同时也有临床资料证明唑来膦酸可以减少乳腺癌患者复发风险^[69]^，但对于肿瘤脑转移患者目前尚缺乏相关足够的临床证据，需要通过进一步的临床试验证实拮抗小胶质细胞/巨噬细胞治疗肿瘤脑转移的效果。

### 2.3 脑转移瘤微血管

肿瘤生长离不开充足的血供，微血管在肿瘤细胞转移机制及生长方面起到重要的作用。脑转移动物模型病理结果显示肿瘤细胞多分布在微血管的周围75 μm左右，距离微血管100 μm的肿瘤细胞多无法生存，Kienast等^[70]^通过荧光示踪法追溯脑转移模型中所有肿瘤细胞的命运，结果发现与血管分离的肿瘤细胞无一例外都走向死亡，Fidler等^[43, 71]^还发现脑转移肿瘤微血管具有平均血管密度（mean vessel density, MVD）低，但管腔多异常扩张不完整的特点。

VEGF是血管形成的关键因子，早前的实验^[72]^已经证明了在脑转移病灶形成过程中，VEGF是必要而非充分的因素。且有研究^[73]^表明脑转移病灶VEGF水平较原发灶高，且与微血管密度（microvessel density, MVD）成正相关。除促血管生成之外，VEGF还可以激活脑转移过程中一部分休眠的细胞，促使其增殖形成微转移灶^[70]^。回顾性分析^[74, 75]^表明使用贝伐珠单抗可以有效地降低肺癌患者脑转移病灶的形成，且并不增高CNS出血风险。然而需要引起注意的是血管形成还受其他因子调控，肺癌脑转移动物实验表明，尽管通过抑制VEGFR可以拮抗VEGF塑造血管的能力，但由于碱性成纤维细胞生长因子（basic fibroblast growth factor, bFGF）的高表达，肿瘤负荷并没有减轻^[71]^。

### 2.4 BBB

BBB是肿瘤细胞形成脑转移病灶首先接触的一个结构，由毛细血管内皮细胞及之间的紧密连接、基膜、AST的树突构成。生理情况下，BBB具有维持CNS稳态的功能，对药物、毒素、离子等物质具有隔离作用。BBB完整性的维持关键是BBB的紧密连接，其紧密连接由跨膜蛋白及周围蛋白构成，跨膜蛋白由封闭蛋白（occludin）、联接黏附分子（junctional adhesion molecules, JAMs）及紧密连接蛋白claudin（BBB上主要为claudin-5）组成，构成细胞之间的相互连接；周围蛋白则分布在紧密连接的两侧，包括闭锁小带（zonula occluden, ZO）及丝状肌动蛋白结合蛋白（afadin）等蛋白，可维持BBB稳定性^[76]^。动物实验^[71, 77]^表明，多种肿瘤细胞株均能顺利通过BBB，且发现直径大于0.25 mm的肿瘤脑转移病灶内部的BBB的完整性受到了不同程度的破坏。肿瘤细胞穿过BBB之间是形成脑转移病灶的第一步，但目前具体机制尚未完全明确。既往Bos等^[78]^通过比较脑转移病灶与原细胞基因表达差异筛选发现环氧合酶（cyclooxygenase-2, COX2）、α2, 6-唾液酸转移酶（α2, 6-sialyltransferase, ST6GALNAC5）及EGF可介导乳腺癌细胞穿过BBB，并推测ST6GALNAC5可以通过促使内皮细胞表面唾液酸化而特异性地介导了脑转移，在结肠癌的患者中，ST6GALNAC5的rs1736858的SNP与脑转移风险高度相关^[79]^。而肿瘤细胞产生的COX2则可以诱导产生前列腺素，从而促进肿瘤细胞高表达MMP1，降解BBB上的Claudin及ZO-1^[80]^。但Lee等^[81]^则认为COX2主要来源并非肿瘤细胞，而为BBB的内皮细胞。神经肽物质P（neuropeptide substance, SP）也可通过改变ZO-1及claudin-5的分布及位置促使肿瘤细胞穿过BBB。体外研究则表明，小细胞肺癌细胞可分泌胎盘生长因子（placental growth factor, PLGF）与VEGFR-1受体结合后，活化ROCK-ERK1/2通路，促使occludin磷酸化而改变BBB的紧密连接，最终易化小细胞肺癌细胞穿过BBB^[82]^。

细胞分泌的囊泡内容物也可介导肿瘤细胞破坏BBB。有研究^[83]^表明，乳腺癌细胞能够直接通过外泌体中的miRNA-105下调紧密连接中的ZO-1破坏BBB的完整性，促使肿瘤细胞向颅内转移，但也有研究则表明BBB的破坏并不仅仅在于紧密连接中的ZO-1蛋白，Tominaga等发现，乳腺癌细胞能分泌的小囊泡（extracellular vesicles, EV），大小约100 nm，包括外泌体及其他的囊泡）中可特定的被BBB内皮细胞摄取，EV中的miRNA181c可抑制BBB内皮细胞上的磷酸肌醇依赖性蛋白激酶1（3-phosphoinositide-dependent protein kinase-1, PDPK1）的表达，而PDPK1下调可使肌动蛋白素（cofilin）磷酸化水平下降并激活cofilin，从而引起肌动蛋白（actin）构象改变，破坏了BBB的紧密连接，促使乳腺癌细胞穿过BBB^[84]^。考虑到肿瘤细胞穿过BBB机制的多样性及其脆弱性，BBB或许并不能作为一个好的抵抗肿瘤入侵CNS及治疗的靶点。此外，既往肿瘤细胞穿透BBB的机制研究主要集中在乳腺癌中，而关于肺癌的研究相对较少，各种肿瘤细胞是否存在不同的机制值得进一步探讨。

而BBB另一成分血管内皮细胞在与CNS转移的肿瘤细胞相互作用主要表现在肿瘤转移过程中的细胞间粘附，在NSCLC细胞脑转移的早期，肿瘤细胞能与内皮细胞通过VLA-4/VCAM-1、ALCAM/ALCAM及LFA-1/ICAM-1粘附，而这些早期粘附分子可作为预防脑转移形成的靶点^[85]^。而另有研究^[86]^则表明脑转移肿瘤NSCLC细胞高表达CD15，并与TNF-α激活的内皮细胞CD62E相互作用，介导肿瘤细胞粘附微血管。此外，肿瘤细胞与内皮细胞相互作用还可促进肿瘤侵袭及肿瘤血管生成，即肿瘤细胞JAK-STAT通路激活后可分泌VEGF，与VEGFR2结合后激活血管内皮的JAK-STAT通路，MMP-9分泌增多，增强肿瘤细胞的侵袭能力^[87]^。

BBB更重要的一点在于它是影响药物治疗脑转移病灶主要的因素。BBB上的内皮细胞丰富表达p-糖蛋白（p-glycoprotein, p-gp）、乳腺癌耐药蛋白（breast cancer resistance protein, BCRP）等ATP结合转运蛋白[ATP-binding cassette (ABC) efflux transporters, ABCG]，ABCG是一类依赖ATP的分子转运体，能将其底物逆浓度梯度转运。目前常用的化疗或靶向药物大多是ABCG家族中某一个或几个的蛋白作用底物，造成CNS病灶药物常常难以达到有效的治疗浓度^[2]^。以靶向药物厄洛替尼为例，在p-gp及BCRP敲除的小鼠中脑组织AUC可达（49.6±3.95）μg/g∙h，而野生型的小鼠脑组织AUC仅仅为（11.0±1.35）μg/g∙h，远低于血浆的（80.2±3.5）μg/g∙h^[88]^。为突破BBB的限制，可采用联合放疗、增大药物剂量及使用ABCG抑制剂等方式，提高药物在CNS中的分布情况以达到良好的治疗效果^[2]^。

### 2.5 脑转移转移瘤微环境的其他细胞成分

其他细胞成分如少突胶质细胞、循环的免疫细胞及CNS间质成分与肿瘤细胞相互作用研究较少。有研究^[89, 90]^表明，将NK细胞种植到乳腺癌或胶质瘤动物模型中，可抑制胶质瘤细胞及HER阳性的乳腺癌细胞生长，但在动物乳腺癌脑转移模型中却发现CD11b阳性的髓样细胞聚集并形成早期肿瘤转移的“土壤”，进一步释放炎症因子S100A8及S100A9，诱导肿瘤细胞趋化^[91]^。甚至在人肿瘤CNS中可见肿瘤相关成纤维细胞（cancer associated fibroblast, CAF），进一步的研究^[92]^表明CAF可促进肿瘤细胞侵袭。

脑转移肿瘤细胞生物学特性的变化及肿瘤细胞与其微环境相互作用可能可以解释肿瘤细胞这颗“种子”如何在颅内“土壤”中定植、生长这一相对低效的转移过程^[93]^，然而，颅脑微环境在脑转移病灶中的肿瘤细胞生物学行为改变中扮演了“筛选”还是“诱导”的角色尚不明确。有研究将肺癌、黑色素瘤与星形胶质细胞共培养后检测全基因组甲基化水平，同样部分复制出脑转移动物模型中甲基化情况的改变，提示星形胶质细胞可以使肿瘤细胞发生甲基化水平变化^[9]^。而McDermott R则提出这样一种模式，星形胶质细胞、小胶质细胞等CNS微环境组分与肿瘤细胞相互作用下可产生某些细胞因子，从而改变肿瘤细胞的miRNA水平，进而影响相应靶基因的表达^[94]^。

总之，恶性肿瘤脑转移问题一直是肿瘤医生关注的热点问题。“种子-土壤”模型的提出为解决脑转移病灶生长提供了一个大的框架。肿瘤细胞与星形胶质细胞、小胶质细胞、微血管、血脑屏障等结构相互作用及肿瘤细胞在中枢神经系统产生的适应性变化，有助于我们发现新的治疗靶点。
